# HiSpike Method for High-Throughput Cost Effective Sequencing of the SARS-CoV-2 Spike Gene

**DOI:** 10.3389/fmed.2021.798130

**Published:** 2022-01-11

**Authors:** Ephraim Fass, Gal Zizelski Valenci, Mor Rubinstein, Paul J. Freidlin, Shira Rosencwaig, Inna Kutikov, Robert Werner, Nofar Ben-Tovim, Efrat Bucris, Oran Erster, Neta S. Zuckerman, Orna Mor, Ella Mendelson, Zeev Dveyrin, Efrat Rorman, Israel Nissan

**Affiliations:** ^1^National Public Health Laboratory, Public Health Services, Ministry of Health, Tel Aviv, Israel; ^2^Central Virology Laboratory, Public Health Services, Ministry of Health, Chaim Sheba Medical Center, Ramat Gan, Israel; ^3^School of Public Health, Sackler Faculty of Medicine, Tel Aviv University, Tel Aviv, Israel

**Keywords:** HiSpike, SARS-CoV-2, NGS, spike variants, variants of concern, high-throughput, cost effective, Omicron variant

## Abstract

The changing nature of the SARS-CoV-2 pandemic poses unprecedented challenges to the world's health systems. Emerging spike gene variants jeopardize global efforts to produce immunity and reduce morbidity and mortality. These challenges require effective real-time genomic surveillance solutions that the medical community can quickly adopt. The SARS-CoV-2 spike protein mediates host receptor recognition and entry into the cell and is susceptible to generation of variants with increased transmissibility and pathogenicity. The spike protein is the primary target of neutralizing antibodies in COVID-19 patients and the most common antigen for induction of effective vaccine immunity. Tight monitoring of spike protein gene variants is key to mitigating COVID-19 spread and generation of vaccine escape mutants. Currently, SARS-CoV-2 sequencing methods are labor intensive and expensive. When sequence demands are high sequencing resources are quickly exhausted. Consequently, most SARS-CoV-2 strains are sequenced in only a few developed countries and rarely in developing regions. This poses the risk that undetected, dangerous variants will emerge. In this work, we present HiSpike, a method for high-throughput cost effective targeted next generation sequencing of the spike gene. This simple three-step method can be completed in < 30 h, can sequence 10-fold more samples compared to conventional methods and at a fraction of their cost. HiSpike has been validated in Israel, and has identified multiple spike variants from real-time field samples including Alpha, Beta, Delta and the emerging Omicron variants. HiSpike provides affordable sequencing options to help laboratories conserve resources for widespread high-throughput, near real-time monitoring of spike gene variants.

## Introduction

Mutations of SARS-CoV-2 spike protein accompanied the transformation of COVID-19 from a local outbreak in Wuhan, China (1–4) into a worldwide pandemic (5–8), inflicting morbidity and mortality on currently over 266 million people with more than 5 million deaths. COVID-19 has severely disrupted health and educational systems, and continues to wreak social, cultural, and economic havoc in afflicted countries. The spike protein, a homotrimeric class I fusion protein, is responsible for the ability of the SARS-CoV-2 virus to recognize and bind to the host cell receptor angiotensin converting enzyme 2 (ACE2) (9–14), and to induce subsequent membrane fusion and viral entry into the host cell (15, 16). It is thought that mutations in the spike protein promoted the initial zoonotic event of coronavirus jump from animal reservoir to human (17–19), and that continued changes of SARS-CoV-2 spike protein in COVID-19 patients promotes increasing adaptation of the virus to the human host (20–22). Indeed, mutations in the SARS-CoV-2 spike protein have resulted in vastly increased transmissibility, infectivity and viral load in humans (6, 7, 23–25).

The spike protein is the target of SARS-CoV-2 convalescent neutralizing antibodies of which over 90 percent target its receptor binding domain (RBD) (26). Therefore, the SARS-CoV-2 spike protein has been selected as the primary target of vaccines (27) and anti-viral drugs (10). Every new mutation in the spike gene is a potential threat to vaccine and drug efficacy. Clearly, the spike protein's crucial roles in both infectivity and susceptibility to neutralization, and its high tendency to mutate make it the most important sequencing target for monitoring circulating viruses.

Now, 2 years into the pandemic, we observe multiple new, predominantly spike protein variants, with superior transmissibility that are changing the dynamics of the pandemic (https://www.who.int/en/activities/tracking-SARS-CoV-2-variants/).

**Figure 1:**
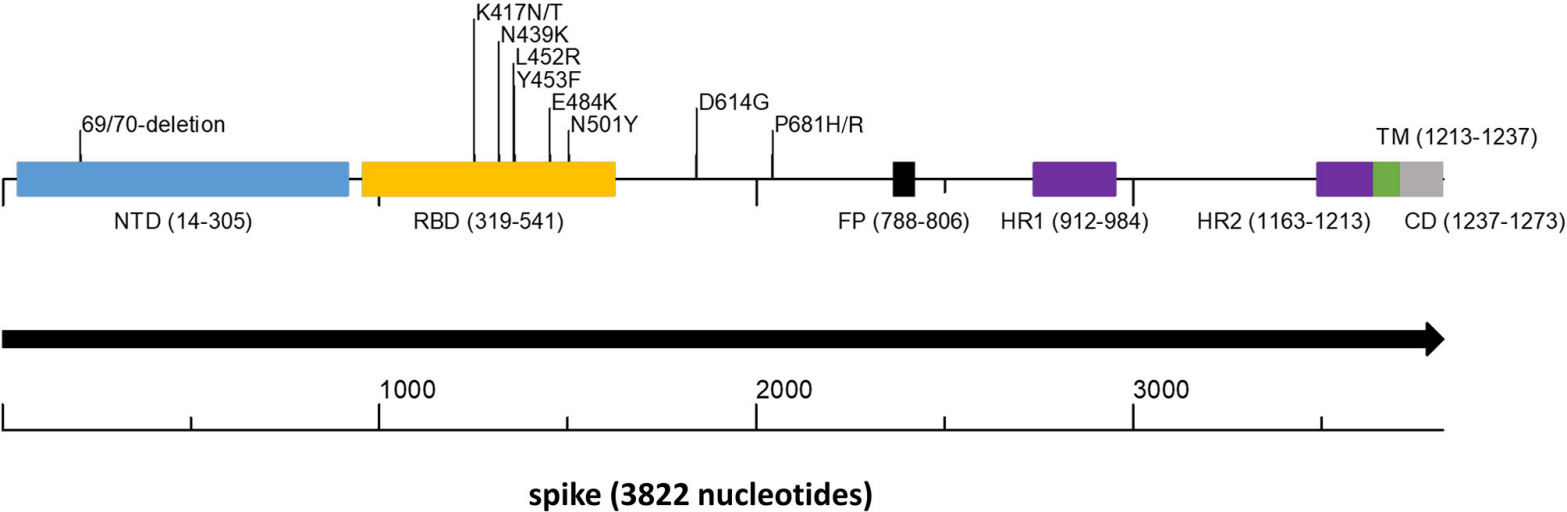
Spike gene scheme with mutations occurring in variants of concern. The spike protein encoded by the spike gene is a large protein of 1,237 amino acids that contains key structural motifs. The figure shows the places of these motifs as well as the location of key amino acid changes that occur in important variants. In parentheses, the start and end of each motif. NTD, N terminal domain (light blue); RBD, Receptor Binding Domain (yellow); FP, Fusion Peptide (black); HR1 and HR2, Heptapeptide Repeat Sequence 1 and 2 (purple); TM, Trans Membrane (green); CD, Cytoplasmic domain (gray).

The SARS-CoV-2 variants Alpha (GISAID GRY, Pango lineage B.1.1.7), and to lesser extent Beta (GISAID GH/501Y.V2, Pango lineage B.1.351) and Gamma (GISAID GR/501Y.V3, Pango lineage P.1) (28–30) dominated worldwide outbreaks during the first half of 2021 (GISAID-NextStrain). The Alpha variant has eight non-synonymous mutations and deletions located in the spike gene (https://virological.org/t/preliminary-genomic-characterization-of-an-emergent-sars-cov-2-lineage-in-the-uk-defined-by-a-novel-set-of-spike-mutations/563). Among these changes are those that significantly affect the binding process to the host cell: N501Y in the RBD, which increases binding affinity to ACE2 (31) and P681H in the S1-S2 furin cleavage site, which was shown to promote entry into respiratory epithelial cells and transmission in animal models (Figure 1). The Beta variant is characterized by eight mutations in the spike protein, including three at critical residues, K417N, E484K, and N501Y, of the RBD (28). Apparently, these mutations evolved independently in the Gamma variant (https://virological.org/t/genomic-characterization-of-an-emergent-sars-cov-2-lineage-in-manaus-preliminary-findings/586). The E484K mutation of these variants has been associated with reduced potency of anti-spike neutralizing antibodies (32). Since June 2021 the Delta variant (GISAID G/478K.V1, Pango lineage B.1.617.2 and all AY sublineages) completely overtook other variants and is leading in prevalence worldwide (GISAID-NextStrain). The Delta variant contains multiple spike protein substitutions (https://www.cdc.gov/coronavirus/2019-ncov/variants/variant-info.html, https://covariants.org/variants/21A.Delta). Specifically, L452R may increase transmissibility by stabilizing the interaction with the ACE2 receptor and reduces neutralization by antibodies, and P681R enhances systemic infection and membrane fusion (https://doi.org/10.1101/2021.06.11.448011, https://doi.org/10.1101/2021.06.17.448820). The Delta variant is considered to be twice as infective and more virulent to unvaccinated patients than all previous variants (https://www.cdc.gov/coronavirus/2019-ncov/variants/delta-variant.html). In November 2021 the world was shocked by the appearance of the heavily mutated SARS-CoV-2 variant Omicron (GISAID GR/484A, Pango lineage B.1.1.529 and Nextstrain clade 21K). The Omicron variant possess more than 30 mutations in its spike gene. Preliminary results indicate higher infection rate than any previous corona variants and increased ability to escape neutralizing antibodies and to infect convalescent and immunized persons (https://www.who.int/news/item/26-11-2021-classification-of-omicron-(b.1.1.529)-sars-cov-2-variant-of-concern, https://www.cdc.gov/coronavirus/2019-ncov/variants/omicron-variant.html).

The joint appearance of multiple spike protein mutations increases concern that new super-spreading variants are on the rise.

In acknowledgment that prompt identification of new variants via genome sequencing is key to outbreak control, emergency public health next generation sequencing (NGS) initiatives have been launched worldwide. The CDC SPHERES (SARS-CoV-2 Sequencing for Public Health Emergency Response, Epidemiology, and Surveillance) program in the US and its ECDC parallel in Europe (European Centre for Disease Prevention and Control. Sequencing of SARS-CoV-2) as well as the COVID-19 Genomics Consortium UK (CoG-UK, https://www.cogconsortium.uk/) are leading examples. A US group has reported monitoring the GISAID (http://www.gisaid.org/) repository database of worldwide SARS-CoV-2 genomic sequences for mutations in the spike gene, associated with frequency shifts at regional and global levels (https://cov.lanl.gov/) (6).

The vast majority of sequences submitted to public depositories originate from Europe and North America (http://www.gisaid.org/). Unfortunately, NGS resources are generally out of reach for developing countries and consequently new variants are likely to evolve undetected from such regions (33). In addition, resources are often stretched even in developed countries, resulting in inadequate coverage.

In this work, we present HiSpike, a simple, high-throughput and cost efficient, 3-step targeted NGS method for full sequencing of the spike encoding gene of SARS-CoV-2. HiSpike can be readily adapted to the workflow of standard laboratories engaged in sequencing. We demonstrate that HiSpike provides results per the spike gene identical to results obtained using the ARTIC protocol, the most used sequencing approach for whole genome sequencing of SARS-CoV-2 (https://artic.network/ncov-2019). We show that HiSpike reliably detects major clade defining mutations in the spike gene including the Delta and Omicron variants. While the ARTIC protocol is an expensive, labor-intensive process, and commonly (for Illumina users) requires 4–5 days, the HiSpike method is generic, high-throughput, easily implemented and obtains sequences in <2 days and at a fraction of the cost.

## Results

To detect on a large scale SARS-CoV-2 variants of high concern, we set out to assemble a sequencing protocol with the following guidelines: The protocol must be high-throughput, accessible to standard laboratories that engage in sequencing, inexpensive with the possibility to use low cost generic reagents, simple, and with a short turnaround time. Because all current and likely future SARS-CoV-2 variants of high concern are based on spike gene mutations (Figure 1) we focused the sequencing to this region.

**Figure 2:**
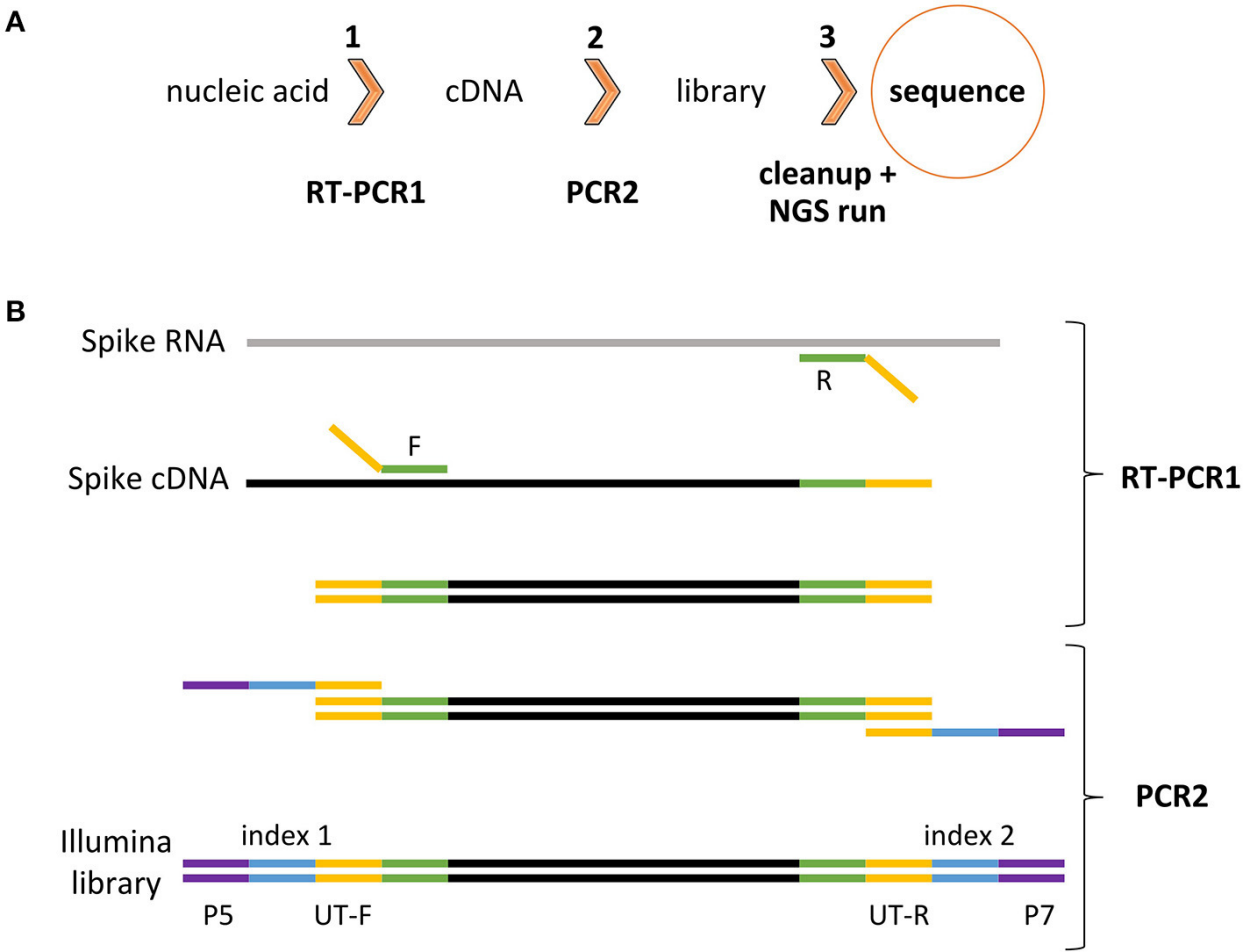
HiSpike method illustration **(A)**. HiSpike three-step protocol outline **(B)**. HiSpike RT-PCR1 and PCR2 Illumina library preparation.

To achieve this goal, the HiSpike method for high-throughput cost effective sequencing of the SARS-CoV-2 spike gene was developed. In the HiSpike method, nucleic acid (NA) samples were converted to spike libraries for sequencing on a MiSeq (Illumina) instrument, the most common NGS sequencing platform used worldwide. The HiSpike method can generate high-throughput sequence data from NA samples within 30 h and involves three simple steps: two consecutive reactions, RT-PCR1 and PCR2, and a single cleanup step of the pooled MiSeq library (Figures 2A,B). The RT-PCR1 converts the viral spike RNA to cDNA, which is amplified using tailed primers to produce spike amplicons with forward and reverse universal tails (Figure 2B). The spike annealing sites of these primers were primarily derived from the established ARTIC V3 primers for tiling PCR amplicons along the SARS-CoV-2 genome with additional 8 primers designed to improve sequencing coverage (Supplementary File 8). Similar to the ARTIC scheme, the RT-PCR1 was conducted in two multiplexed reactions to produce two overlapping sets of ~400 bp spike gene amplicons enclosed by forward and reverse universal tails. These amplicons were combined and subjected to PCR2, which added unique dual indexes and the Illumina P5 and P7 flow cell adaptors to each sample (Figure 2B).

A series of experiments were initially done to optimize and simplify reaction conditions, primer properties, and enzymatic settings to robustly produce high quality libraries by the HiSpike method (data not shown). The optimized RT-PCR1 conditions eliminated the need for equilibration between samples due to the limited primer levels (0.045 μM) that were maxed out during the 45 PCR cycles. Consequently, similar dsDNA amplicon levels in each sample were found and roughly maintained throughout the sequencing process (An example of a MiSeq sample read distribution is shown in Supplementary File 1). In this study, all HiSpike libraries were sequenced with MiSeq's smallest and fastest flow cell the V2 Nano kit. In a typical 250-bp paired-end run, we obtained about 2 million reads and could simultaneously sequence 96 samples.

For preliminary assessment of the HiSpike sequencing results, four samples representing different variants (S-0007, S-0031, S-0043, and S-0044, Supplementary File 2), also were sequenced using the traditional Sanger method. The Sanger primers were designed to generate six overlapping PCR fragments that cover the entire spike gene (Supplementary File 3). The sequences obtained using HiSpike and Sanger were identical. Next, to assess the sequencing performance of the HiSpike method, we compared its sequence results to spike gene sequences obtained with the established ARTIC whole genome sequencing method which has been recommended by leading international organizations such as the WHO and ECDC (https://www.who.int/publications/i/item/9789240018440 and https://www.ecdc.europa.eu/sites/default/files/documents/Sequencing-of-SARS-CoV-2-first-update.pdf). Both methods were used to compare 90 positive SARS-CoV-2 samples. These samples spanned a variety of viral loads as indicated by their Ct values, as measured in a real-time RT-PCR assay. The HiSpike method was much simpler to perform, with significantly shortened time to acquisition of results and greatly reduced cost (respectively, Supplementary Files 4, 5).

**Figure 3:**
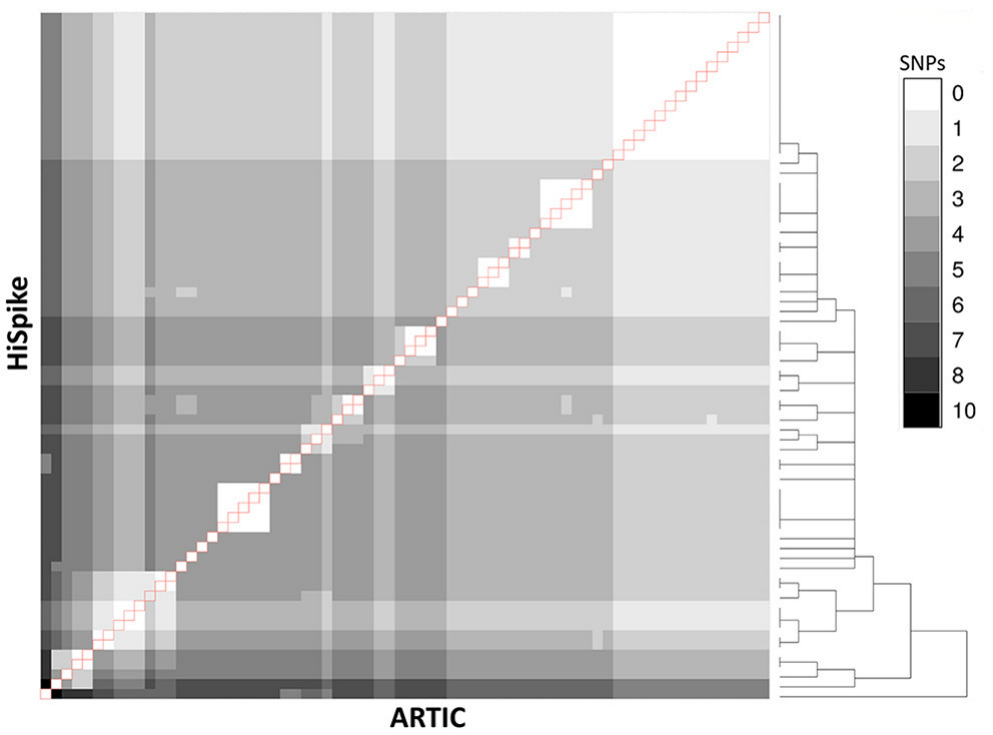
Comparison between ARTIC and HiSpike methods. Spike gene sequences of 70 samples (with > 90% spike sequence coverage in both ARTIC and HiSpike methods) were compared using a heat-map. Gray shades between white and black indicate 0 to 10 SNPs respectively. HiSpike sequences were aligned based on a hierarchical clustering tree (shown to the right) vertically and the ARTIC sequences of the same samples were aligned horizontally to the HiSpike samples. Pairs of the same sample are represented in the diagonal line (outlined in red). Sample 11 (counting from the left) exhibits a single SNP difference between the HiSpike and Artic results; all the other samples show identical sequences.

**Figure 4:**
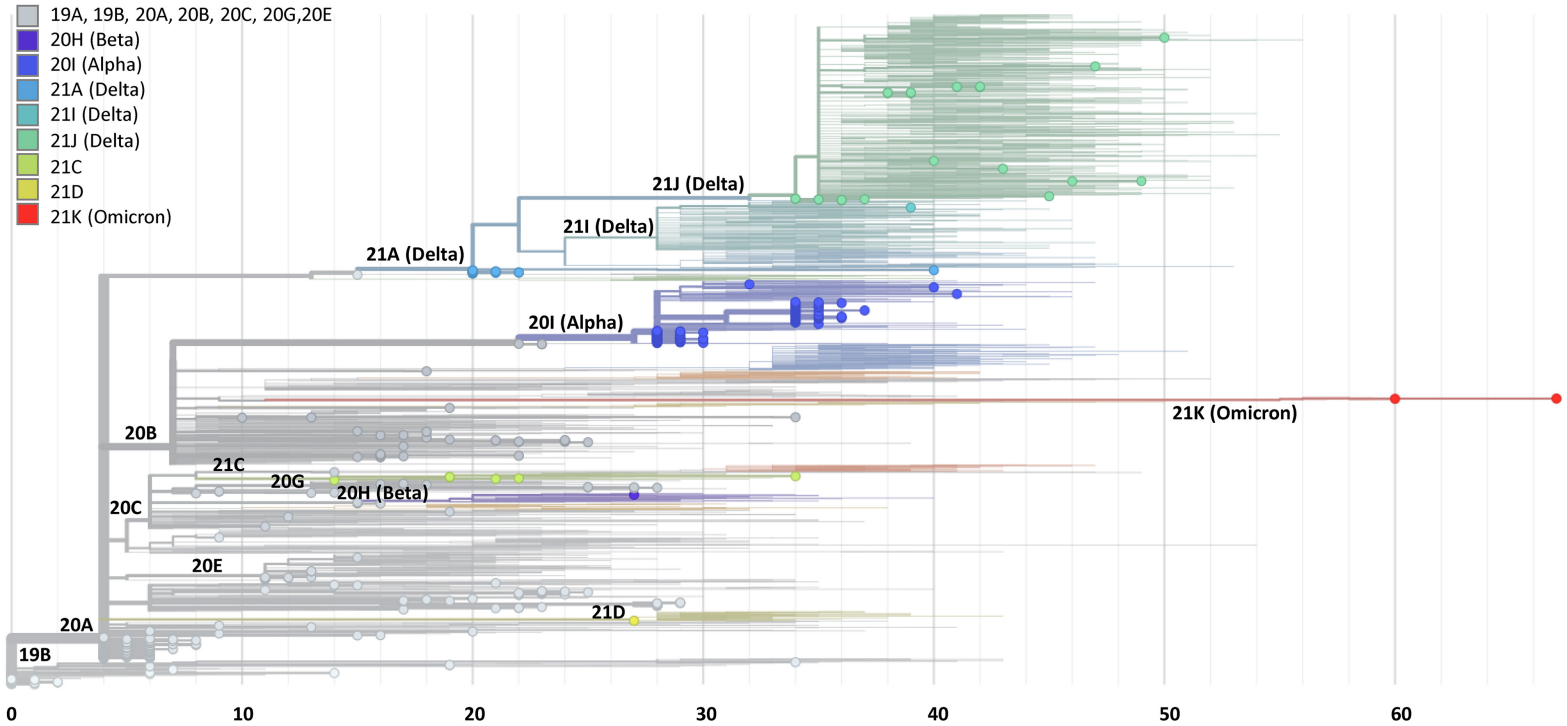
Phylogenetic tree based on spike gene sequences generated by the HiSpike method. Spike gene sequences of 441 samples generated by the HiSpike method were uploaded to the Nextclade platform. SARS-CoV-2 clades are illustrated by different colors and locations on the rectangular tree. The X axis indicates the number of mutations relative to the reference full viral genome. HiSpike sequences are represented by the full circles on a background of the Nextclade's global representative clade tree of December 8 2021.

Among the 90 samples, 88 showed identical sequences. Only two samples showed a single nucleotide difference. These differences appeared in samples with relatively high Cts (28 and 30), indicating low levels of viral NA in the original samples. The sequence coverage of 70 samples was > 90% in both methods and an overall visualization of their spike gene sequences identity level is shown in Figure 3. The white diagonal line represents full spike gene identity (0 SNPs) between pairs of the same samples sequenced by HiSpike and ARTIC methods. The spike sequences generated by HiSpike differentiated various clades (Figure 4; Supplementary File 2) into Nextclade branches that were similar to those assigned via ARTIC V3 whole genome sequencing. Notably, similar high spike gene coverage in both the ARTIC and HiSpike methods was observed for most of the samples (Supplementary Files 6A,B).

The validated HiSpike sequencing capacities and short turnaround time from NA extraction to spike gene sequence acquisition led to the selection of this method in Israel for nationally urgent sequence assignments of positive COVID-19 cases. These included travelers arriving from countries with a high incidence of spike variants of concern, instances of reinfection, severely ill patients, and hospitalized pregnant women. A total of 306 clinical positive SARS-CoV-2 NA samples arrived on five different occasions during 2 critical weeks in January (January 14 to 28, 2021). During this time, a global spread of variants of concern (e.g., Alpha, Beta, and Gamma) forced lockdowns in many countries including Israel, while massive COVID-19 immunization efforts took place. Samples were processed upon arrival and the average time from sample to sequence was <30 h. The HiSpike method identified 119 Alpha variants and 7 Beta variants among other multiple clade branches (Figure 4; Supplementary File 2).

**Figure 5:**
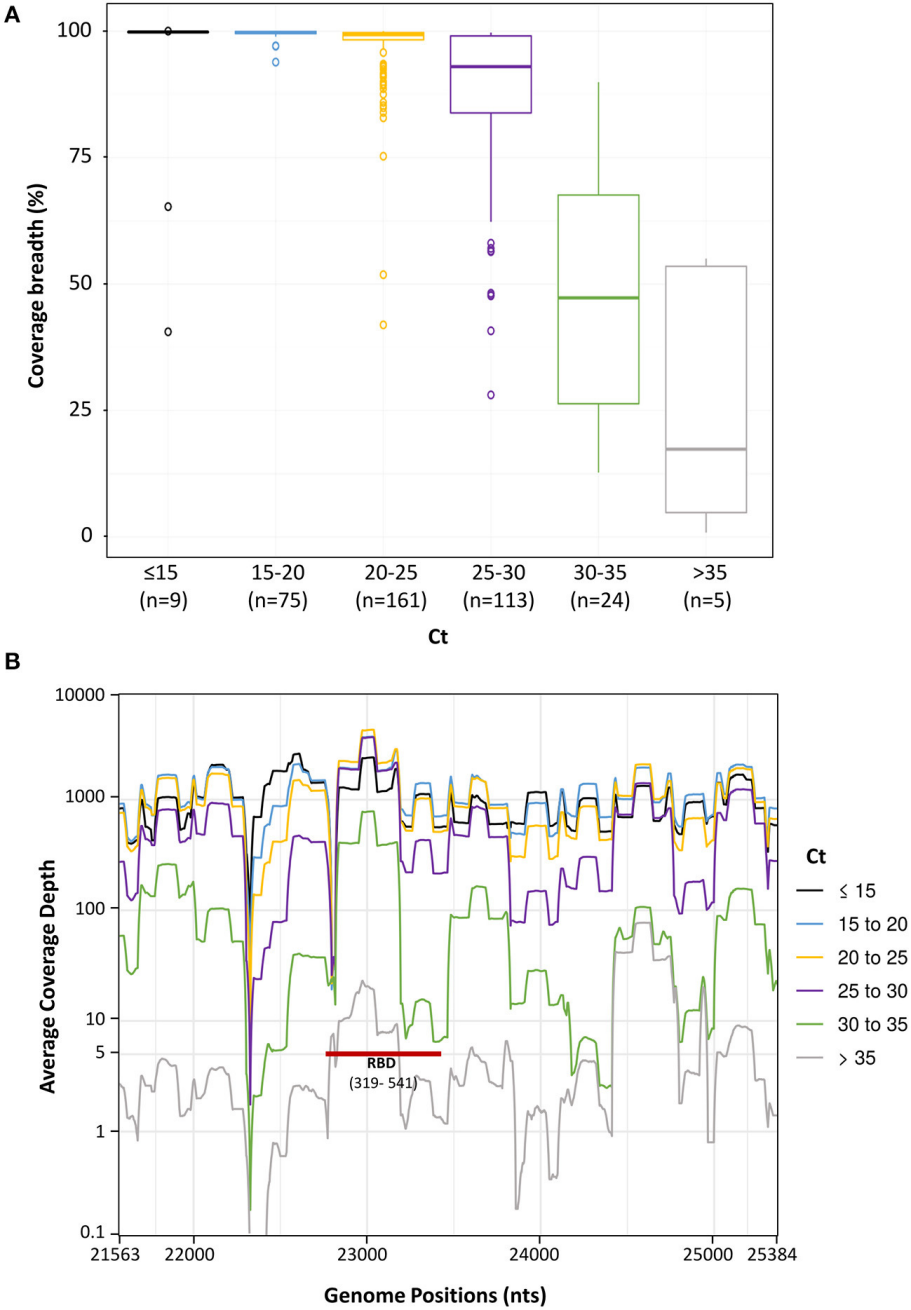
Spike sequence breadth and depth as a function of different sample Ct levels. Spike gene sequences of 396 samples were divided to 6 groups based on their Ct values (up to 15, 15–20, 20–25, 25–30, 30–35, and over 35). The number (n) of sample in each group is in brackets. **(A)**. The percentage of spike gene coverage, with at least 5 reads, at different Ct levels is shown by Boxplots. The Boxplots represent the coverage breadth value distributions from the lower to the upper quartiles. The inner horizontal line indicates the median. The vertical whiskers extend to the most extreme data point which is no more than 1.5 times the interquartile range. More extreme data points are drawn as circles. **(B)**. Sequence coverage depth along the spike gene. The average coverage depth was plotted, on a logarithmic scale, along the genome positions. Lines were smoothed by a sliding window of length 20. At coverage depth x5 the spike gene region encoding the receptor binding domain is marked with a red line and annotated (RBD and in brackets its amino acid location).

To determine HiSpike's limit of detection, we plotted the Ct value (as the viral load indicator, determined by real-time RT-PCR) and spike gene sequence coverage breadth. To this end, we analyzed 396 samples comprised of the validation sample set and the urgent clinical samples. These samples showed Ct values ranging from 11 to 40. The median coverages of samples were as follows: for Ct 11 - 25, Ct 25–30, Ct 30–35, and Ct 35−40, respective median coverages were 99.3, 93.0, 47.3, and 17.3% (Figure 5A). Notably, samples in this study were collected from several clinical laboratories that use various extraction systems and SARS-CoV-2 determination methods. In rare cases, we observed low coverage in samples with Cts below 25 (Figure 5A) which likely represented poor quality or degraded NA, and in our experience similar low coverage of these samples was also apparent when using the ARTIC method.

We next assessed the average coverage depth along the spike gene with relation to its Ct. This parameter is important because it provides insights into the coverage expectancy along the spike sequence. As seen in Figure 5B, the average coverage depth along the spike gene varied. At Cts of 25–30, the average coverage depth was more than 100. At Cts above 30 there was a significant decline and more regions remained below the five independent reads cut off (Figure 5B). Notably, key parts of the RBD in the spike gene were amplified and sequenced at coverage of 5 reads or more even in samples with high Cts (over 30), allowing detection of mutations like N501Y (Figure 5B, RBD red bar).

These results indicate that the HiSpike method is highly robust and suitable for full spike gene sequencing of samples with Ct values up to 30. This is similar to the specifications previously shown for ARTIC based amplicons (34) and other SARS-CoV-2 sequencing methods (35). Importantly, key regions in the spike gene such as the RBD (Figure 5B) will likely be detected also in samples with very low virus content.

In June 2021 Delta variant overtook all previous variants and become the worldwide dominant corona variant. In November 2021, the heavily mutated Omicron variant emerged. To assess the performance of HiSpike method to sequence contemporary variants, we performed an *in-silico* analysis of the primer scheme followed by an *in-vitro* confirmation. The RT-PCR1 HiSpike primers (Supplementary File 8) were tested *in-silico* for matching to current SARS-CoV-2 variants. The matching was performed on 286 current GISAID sequences (of sampling dated 15 - 30 November 2021) that included different Delta variants (clades 21A, 21I, 21J) and the newly emerging Omicron variant.

The analysis showed that 39 (out of 42) primers maintain perfect alignment with most of the variants (Supplementary Files 11, 12). Three primers termed: 3_F1_HiSpike, 4_R_HiSpike, and 11_F_HiSpike showed mismatches to a large number of the tested sequences (Supplementary File 11). Primer 3_F1_HiSpike showing a single mismatch to ~43% of the sequences has a backup primer 3_F2_HiSpike and is not expected to affect the assay performance. Primer 11_F_HiSpike showed a single mismatch to the majority of the sequences. Because this mismatch is 7 bases upstream to its 3' end, we do not expect it to significantly affect its annealing capability and elongation of its target. Primer 4_R_HiSpike showed 8 mismatches to the majority of the sequences all of which located 15 bases upstream to its 3' end and, therefore, the annealing of this primer may be affected. Nevertheless, 4_R_HiSpike is a reverse primer and therefore, participates in the initial cDNA generation step that is performed at 45°C. We expect that at this low temperature this primer will preserve its ability to bind and participate in cDNA synthesis. Once this primer binds, all following PCR steps should have a full binding site on the cDNA.

Next, we validated the performance of the HiSpike method *in-vitro* on a collection 42 contemporary SARS-CoV-2 samples. These samples included 40 Delta variant sub lineages (clades 21A, 21I, and 21) and two Omicron variants (clade 21K). The HiSpike results showed the coverage breadth of the sample set was in correlation to the sample viral load. Samples with Cts between 20 and 25 showed an average coverage breadth of ~ 88 %. Notably, HiSpike demonstrated a sequence coverage that was above 95 % for all different representatives of the Delta variants and most importantly for the emerging Omicron variant. As observed with earlier variants, also in the current variants the samples with lower viral loads (Cts above 25) showed a decline in coverage breadth. Nevertheless, even in these samples we obtained enough sequence information to detect the variant's mutation signatures and determine clades with the nextclade web tool (Supplementary File 13; Figure 4). Together, the *in silico* and *in vitro* results show that in spite of SARS-CoV-2 evolution the HiSpike method maintains its relevance as a powerful tool for spike gene sequencing.

## Materials and Methods

All methods were performed in accordance with the relevant guidelines and regulations: all samples used in this study were collected in accordance with the Public Health Ordinance of the State of Israel and directives of the Israel Ministry of Health (325589721 and 485090420).

The study has been approved by the Institutional Review Board of the Sheba Medical Center #7045-20-smc. The approval covers using remains of clinical samples for assessing new approaches for diagnosis of SARS-2 and waives the need for informed consent. We hereby state that our laboratory has permission to receive remains of SARS-CoV-2 samples from source laboratories.

For user convenience we have included an instruction manual for implementing the HiSpike method in Supplementary File 7.

### Sample Source and Characterization

Positive COVID-19 samples of either primary nasopharyngeal swabs or extracted NA were collected from various COVID-19 laboratories in Israel. NA samples were stored at −80°C prior to use and primary samples were kept at 4°C (up to 72 h) prior to extraction. Viral inactivation of swab samples was performed using Lysis Buffer (Seegene) at a buffer to sample volume ratio of 0.75 (200 μl buffer: 267 μl sample) for 10 min at room temperature. From total volume of 400 μl, 50 μl of NA samples were extracted using automated instruments of MagNA Pure (Roche) or magLEAD (Precision System Science Co., Ltd.).

The cycle thresholds (Cts) of SARS-CoV-2 positive samples were determined either by the source lab or, in unknown cases, in house using Allplex 2019-nCoV qPCR kit (Seegene). The qPCR reactions were performed according to the manufacturer's instructions. Briefly, reactions were comprised of 25 μL, consisting of 17 μL master mixes (Buffer, RNase-free water and 2019-nCoV MOM and Enzyme) and 8 μL NA templates. Reactions were added to 96-well plates each of which included a positive and a negative control. The qPCRs were performed on a CFX96 Touch instrument (Bio-Rad) with the following conditions: 20 min reverse transcriptase step at 50°C, 15 min heat-start step at 95°C, and 45 cycles of 15 second denaturing period at 95°C followed by a combined annealing and extension step at 58°C for 30 s. Finally, the Ct values of E, RdRP, and N genes were determined using the Seegene Viewer software (Seegene). For simplicity, in this work we used the average Ct of all three genes.

### HiSpike Library Preparation

Primers used in this study were manufactured by metabion or IDT and are shown in Supplementary Files 3, 8, 9.

RT-PCR1 primers contained 3' annealing sequences corresponding to sites along the spike gene and 5' forward or reverse universal tails (denoted in the sequence below by upper case letters). A primer binding site, an Illumina component for initiation of reads 1 and 2 (denoted below with lower case letters), was located between the universal tails and the annealing regions resulting in the following strands: 5'TCGTCGGCAGCGTCagatgtgtataagagacag[spike annealing sense sequence]3'and 5'GTCTCGTGGGCTCGGagatgtgtataagagacag[spike annealing antisense sequence] 3' for the forward and reverse primers respectively (Supplementary File 8). The spike annealing sites (indicated in square brackets) were mostly derived from the SARS-CoV-2 V3 ARTIC primers for tiling amplicons (https://artic.network/resources/ncov/ncov-amplicon-v3.pdf) and in some cases, additional primers with alternative annealing sequences were added to increase the sequencing coverage (Supplementary File 8).

The PCR2 primers termed F-P5 (i01–i12) and R-P7 (i01–i08) contained the following sequences: 5'AATGATACGGCGACCACCGAGATCTACAC[i5]tcgtcggcagcgtc3' and 5'CAAGCAGAAGACGGCATACGAGAT[i7]gtctcgtgggctcgg3' respectively (Supplementary File 9). Each primer in the primer pair harbored a unique 8 base index, denoted i5 or i7, and a 3' sequence (in lower case letters) of the forward and reverse universal tails that anneal and amplify the RT-PCR1 products, thereby adding the dual index and unidirectional P5 and P7 termini.

#### Step 1 of the HiSpike Method-Library Preparation: RT-PCR1

Two 10 μL multiplex RT-PCR1 reactions were composed of either primer mix 1 or mix 2 using SensiFast (Meridian Bioscience). The reaction consisted of 5 μL 2x SensiFAST, 0.2 μL RiboSafe RNase Inhibitor, 0.1 μL Reverse transcriptase, 0.7 μL H_2_O, and 1 μL of primer mix set 1 or 2 (0.45 μM of each primer), and 3 μL NA template. The RT-PCR was performed on a Biometra TOne 96 Standard Thermal Cycler instrument (Analytik Jena) with the following conditions: 10 min reverse transcriptase step at 45°C, 4 min heat-start step at 95°C, and 45 cycles of 15 s denaturing period at 95°C, annealing of 30 s at 60°C and elongation at 72°C for 30 s. The products of corresponding samples from reaction sets 1 and 2 were combined and used as the template for PCR2.

#### Step 2 of the HiSpike Method - Library Preparation: PCR2

PCR2 was performed using KOD Hot Start DNA Polymerase (Merck Millipore). Reactions were set to 15 μL and contained 4 μL of each of the uniquely indexed primers F-P5 (1.2 μM) and R-P7 (1.2 μM), 1.0 μL RT-PCR template, 1.5 μL 10X Buffer, 1.5 μL dNTPs (2 mM each), 1.5 μL MgSO_4_ (25 mM), 1.2 μL H_2_O, and 0.3 μL KOD Hot Start DNA Polymerase (1 U/ μL). Reactions were performed on a Biometra TOne 96 Standard Thermal Cycler instrument (Analytik Jena) with the following conditions: 4 min hot-start step at 95°C, and 15 cycles of 15 s denaturing period at 95°C, annealing of 30 s at 58°C and elongation at 72°C for 30 s.

### Library Cleanup and MiSeq Loading (Step 3 of the HiSpike Method)

Products of PCR2 were pooled by collecting 4 μL from each well. The pooled library was diluted 1: 3 (sample: H_2_O) and purified using the ProNex Size-Selective Purification System (Promega) at a ratio of 1.4: 1 (ProNex chemistry: sample) according to the manufacturer's instructions. In Short, 70 μL beads and 50 μL diluted sample were mixed and incubated for 10 min and placed on a magnetic stand for 2 min. The supernatant was discarded and the resin was washed twice by two consecutive 1-min washes using 200 μl of Wash Buffer each. After discarding the second portion of wash buffer, the resin was allowed to air-dry for 5 min. It was then removed from the magnetic stand and resuspended with 50 μL elution buffer for 5 min. Finally, the sample was placed on the magnetic stand for 1 min and the eluted purified HiSpike library was collected. The dsDNA concentration of the purified HiSpike library was determined by Denovix QFX Fluorometer, using the DeNovix dsDNA High Sensitivity assay.

The HiSpike library, with an expected fragment average size of 550 bp, was examined by agarose gel electrophoresis (1.7%) and on the Fragment Analyzer 5200 (Agilent) with HS NGS Fragment (1–6,000bp) kit. The purified library was diluted to 4 nM and denatured by mixing 5 μl of library with 5 μL 0.2 N NaOH for 5 min. The denatured library was further diluted to 12 pM and a 1% PhiX control (PhiX Control v3) was added. Sequencing of all HiSpike samples in this study were performed on an Illumina MiSeq platform using a 250-bp paired-end read v2 Nano kit (Cat. # MS-103-1003). For user convenience, we included a typical MiSeq sample sheet for 96 indexes in Supplementary File 10.

### Sanger Sequencing

To obtain the full sequence of the spike gene (3822 bp) six sets of primers were designed (Supplementary File 3). Amplicons, with overlapping regions of ~100bp, were generated using PCRBIO GO One-step RT-PCR kit (Cat. # PB10.53-10) according to the manufacturer's instructions and sequenced using ABI 3500 Bioanalyzer. Data analysis was performed using the Geneious software package (https://www.geneious.com/).

### ARTIC V3 Protocol for SARS-CoV-2 Full Genome Sequencing

RNA in extracted NAs was reverse transcribed to single strand cDNA using SuperScript IV (ThermoFisher Scientific, Waltham, MA, USA) as per manufacturer's instructions. SARS-CoV-2 specific primers designed to capture SARS-CoV-2 whole genome (version 3, https://github.com/artic-network/artic-ncov2019/blob/master/primer_schemes/nCoV-2019/V3/nCoV-2019.tsv) - total 218 primers, divided into two primer pools designed by Josh Quick from ARTIC Network) were used to generate double strand cDNA and amplify it via PCR using Q5 Hot Start DNA Polymerase (NEB) (36). Briefly, each sample underwent two parallel PCR reactions with primer pool 1 or 2 and 5X Q5 reaction buffer, 19 mM dNTPs and nuclease-free water. The resulting DNA was combined and quantified with a Denovix QFX Fluorometer, using the DeNovix dsDNA High Sensitivity assay as per manufacturer's instructions, and 1ng of amplicon DNA in 5 μL per sample was used for the library preparation. Libraries were prepared using the NexteraXT library preparation kit and NexteraXT index kit V2 as per manufacturer's instructions (Illumina, San Diego, CA, USA). Pooled libraries were purified with AMPure XP magnetic beads (Beckman Coulter, Brea, CA, USA) and the pooled library concentration was determined by Denovix QFX Fluorometer, using the DeNovix dsDNA High Sensitivity assay. The pooled library validation and mean fragment size was determined by Fragment Analyzer 5200 (Agilent) with the HS NGS Fragment (1-6,000 bp) kit. The mean fragment size was ~350 bp, as expected. The molarity concentration of the library was calculated and diluted to 4 nM, denatured and further diluted to 12 pM. Finally, the sample was loaded on MiSeq Reagent Kit v3 (600-cycle, Cat. # MS-102-3003) and a paired end 151X2 program was set.

### Sequence Clean-Up, Mapping and Determination of Coverage (Bioinformatics Analysis)

Trimmomatic-0.36 was used for quality trimming of the raw read sequences, by clipping 3' read ends with sliding window quality lower than 15 in a window of 4 nucleotides, and at the same time trimming the 5' primer sequence used for targeting the virus genome, by cropping 30 nucleotides from the head of the read. Reads with < 50 bases remaining length were filtered out. The resulting reads were mapped to the spike gene of the Wuhan-Hu-1 reference genome (NCBI Reference Sequence NC_045512.2) using bwa v0.7.12-r1039 MEM algorithm. Samtools 1.10 was used for sorting, merging pileup, and consensus sequence was created with ivar 1.0 with minimum quality score threshold to count base of 15, and minimum depth to call consensus of 5. Point coverage depth was determined with samtools depth with base quality threshold of 15 and mapping quality threshold of 15. Coverage breadth was defined as the ratio of the reference sequence covered by X5 depth or more. FASTA sequences were uploaded to Nextclade (https://clades.nextstrain.org/). Nextclade is a web-tool that identifies differences between reference and user uploaded genomes. For each uploaded sequence, Nextclade provided a complete list of established and new spike gene mutations, both in nucleotide and amino acid notation.

### *In*-*silico* Primer Match Testing

Two hundred and eighty six high quality SARS-COV2 genome sequences, with <20 Ns, and patient information with collection dates between November 15 to 30 2021, were downloaded from GISAID database (https://www.gisaid.org/). The R package “Biostrings” was used to find the HiSpike primer annealing regions in the genome sequences, allowing none to 6 mismatches. Supplementary File 11 shows for each primer, in how many genomes it was found with perfect match, in how many it was found with either 1, 2, 3, 4, 5, or 6 mismatches, and in how many genomes it was not found when allowing maximum 6 mismatches. Supplementary File 12 shows for each SARS-COV2 clade, the average number of primer sequences that match genomes belonging to this clade with perfect match, the average number primers that match with 1, 2, 3, 4, 5, or 6 mismatches, and the average number of primers that are not found when allowing maximum 6 mismatches, in this strain's genomes.

### Data Availability

The HiSpike generated sequences are available to the public at the NCBI BioProject number PRJNA751747.

## Discussion

The SARS-CoV-2 virus is likely to continue to evolve over time in human populations. Real-time monitoring of the circulating strains by sequencing is essential to combat the current pandemic. The spike protein centrality in viral infectivity, transmissibility, and reactivity with neutralizing antibodies is currently the most important element for sequence monitoring. Globally, multiplex tiling PCR methods, such as ARTIC, on Illumina platforms are leading the full genome sequencing of SARS-CoV-2. While serving as an essential tool for genomic epidemiology, these methods are relatively time consuming, labor intensive and expensive, and consequently their use is limited for many laboratories, and out of reach in developing countries (33). The HiSpike method is an attractive option to fill in this gap. Using this method, one can produce high quality sequencing libraries in three short steps, RT-PCR1, PCR2, and library cleanup making the assay simple and inexpensive (Supplementary Files 4, 5).

The RT-PCR1 primer annealing sites along the spike gene were derived from the well-established ARTIC primers (https://www.protocols.io/view/ncov-2019-sequencing-protocol-v3-locost-bh42j8ye), plus 8 in-house newly designed primers to achieve adequate coverage. The addition of these in-house newly designed primers improve the coverage breadth and method robustness as demonstrated by the *in-silico* analysis (Supplementary File 11) and successful sequencing of all tested variants, including various Delta clades and the emerging Omicron. Due to the continuous evolution of SARS-CoV-2, primers for detection and sequencing methods must be reassessed periodically and updated as necessary.

The 5' end of HiSpike primers contained for simplification and streamlining, forward and reverse universal tails. The tail design of RT-PCR1 and PCR2 HiSpike primers shares similar characteristics with our patent pending method (37). Notably, Gohl et al. (33) also incorporated these adapter tails to ARTIC V3 primers for full genome sequencing. Although, Gohl's method required four PCR pools instead of the original ARTIC two pools, it significantly reduced the cost and labor of classic ARTIC methods. Here we show that HiSpike is a much simpler method that: (1) requires (similar to original ARTIC version) only two pools per sample, (2) generates cDNA and target amplification in a single RT-PCR1 step, and (3) does not require library normalization because it is achieved by substantial consumption of the limited levels of PCR1 primers. HiSpike is also flexible, as it can be performed with generic reagents that are widely available from various manufactures (Supplementary File 5). In this study, we used SensiFAST SYBR Hi-ROX Kit (Cat. # BIO-92005) and KOD Hot Start DNA Polymerase (Cat. # 71086) for RT-PCR1 and PCR2 respectively. Yet, high quality libraries also were obtained with Xpert One-Step RT-PCR Kit (grisp Cat. GE50.0100) for RT-PCR1 and TaKaRa Ex Taq (TaKaRa Cat. # RR001A) for PCR2 (data not shown). The simplicity and reagent flexibility together with its low cost make HiSpike significantly more accessible and suitable for a wider community.

Library preparation is the major cost input for SARS-CoV-2 sequencing. Therefore, the new library preparation protocol that we have implemented for HiSpike, significantly cuts this cost. For instance, in Israel, while library preparation of 96 samples using ARTIC method cost 4,516 USD, using HiSpike the method cost 81 USD (Supplementary File 5). HiSpike further cuts the sequencing cost since the spike gene represents only 12.8% of the SARS-CoV-2 genome, allowing the load of many more samples on the same flowcell. This also makes the method remarkably useful for high-throughput sequencing. The HiSpike method can adequately sequence 96 samples using the cheapest and fastest MiSeq's Nano kit. In a typical run, we obtained about 2 million reads. The MiSeq Reagent Kit V2 (500-cycles) (Cat. # MS-102-2003) offers more than 10 fold reads per run (24–30 million). Therefore, at the same cluster density, using this kit, it is possible to sequence nearly 1000 samples per flow cell. Other whole genome sequencing methods ((34), and ARTIC V3) require the most expensive MiSeq Reagent Kit V3 (600-cycle) (Cat. # MS-102-3003) that gives 44–50 million reads per run. Using HiSpike we can expect to sequence with necessary and sufficient clade and variant detection accuracy, up to 2,400 samples in a single MiSeq Reagent Kit V3 run.

According to GISAID data nearly 80% of the deposited SARS-CoV-2 whole genome sequences were generated using Illumina platforms. These methods have proven most effective for high resolution epidemiologic purposes in which short time to sequence and high volume is not the primary desire. Using NovaSeq instruments (Illumina) coupled with robotic liquid handlers for library preparation, laboratories are now able to significantly increase throughput and reduce the processing time. However, these are highly demanding resources. Another option for shortening time to results is the Oxford nanopore technique (ARTIC's original method) which can achieve sample to sequence in <24 h. However, this method is not high-throughput and its high price per sample is prohibitive for many laboratories. Proof of concept for a more affordable sequencing approach using the Oxford nanopore technique has been recently published (https://doi.org/10.1038/s41598-021-95563-w). This study, which cites our HiSpike method, adopted the same philosophy of sequencing the spike gene alone.

These methods enable low resource laboratories to perform high volumes of samples in a short time.

As indicated, for the HiSpike method we chose to use the smallest and cheapest nanorun MiSeq kit. Consequently, compared to ARTIC we obtained a slightly lower coverage for low viral loads samples (Supplementary File 6A). In such samples, the coverage deficiencies were focused at four positions, nucleotides 755–775, 1226–1235, 2257, and 3739–3747 (Supplementary File 6B). Nevertheless, these positions did not encompass the key spike gene regions such as the RBD (Supplementary File 6B), and only one position fell within the RBD but has not to date been involved in a mutation defining a variant of concern.

Low viral load may result from various reasons including low sample quality, poor nucleic acid extraction yield, inadequate storage or transportation and more. In these cases it is recommended to resample the patients and possibly increase viral concentration before sequencing. In many cases low viral load indicates post COVID19 status in which remnants of fragmented nucleic virus material can be detected by PCR for weeks. Such fragmented material is extremely difficult to sequence, makes it impossible to receive full genome coverage. As a policy, the Israeli Corona sequencing consortium consider samples with Cts above 33 inadequate for sequencing using the commercial Illumina COVIDseq method.

Our main focus for HiSpike development, was to offer a fast, simple and cheap alternative for SARS-CoV-2 sequencing. Therefore, by intention we selected the smallest kit of MiSeq sequencer (nanorun). However, we speculate that for low viral load sample the user can dedicate more reads per sample. We observed this phenomena by sequencing the same library of 45 samples twice on the smallest MiSeq sequencer kit (unpublished data). The coverage breadth of the combined runs for samples with high Cts (above 25) was significantly higher than each run alone indicating, that increasing the reads (flow cell space) will increase sequence coverage breadth. Therefore, in specific cases where higher sequence coverage of low viral load samples (Ct > 25) is desired one may choose to use a larger sequencing kit or reduce the number of samples per flow cell.

Methods that require less reads per sample, also save expensive storage space and computation resources for analysis. Essential web platforms like GISAID (https://www.gisaid.org/) allow submission of complete or nearly complete genomes, while they also perform multiple analyses that are focused on the spike gene such as spike protein mutation surveillance. However, a much needed spike dedicated database for sequence deposit and global sharing for comparison and variant alerts, has yet to be established.

We have enumerated the strengths of HiSpike and its desirable features and benefits. Nevertheless, HiSpike has limitations, chief among them being that it does not sequence genomic regions outside the spike gene, which are covered by other protocols for whole genome sequencing (34, 38–40). To the best of our knowledge, mutations outside the spike gene that affect transmissibility are not common. The most important aspect of this limitation is that molecular epidemiology including its uses for high-resolution clade definition and outbreak profiling to aid contact tracing, is mostly effective at the whole genome level.

As far as we know, HiSpike is the first validated tool for specifically sequencing the spike gene to monitor mutations of high concern in clinical samples. To ease transitions to the HiSpike method, we included a detailed user instruction manual (Supplementary File 7). We propose using HiSpike for routine sequencing of positive SARS-CoV-2 samples for near real-time monitoring of emerging spike mutations. In this manner, public health resources can help contain potential super spreading or vaccine-escape variants, even before these variants cause frequency shifts at the local, regional, or global levels.

## Data Availability Statement

The datasets presented in this study can be found in online repositories. The names of the repository/repositories and accession number(s) can be found in the article/Supplementary Material.

## Ethics Statement

The studies involving human participants were reviewed and approved by Institutional Review Board of the Sheba Medical Center #7045-20-smc. Written informed consent for participation was not required for this study in accordance with the national legislation and the institutional requirements.

## Author Contributions

EF and IN: conceived the HiSpike concept, conceived and designed the experiments, conducted experiments, analyzed data, and wrote the manuscript. GV: conceived and designed the experiments, conducted experiments, and helped write the manuscript. MR: analyzed data and helped write the manuscript. PF: wrote much of, and critically reviewed, the manuscript. SR: critical reading and writing of the manuscript. IK, RW, and NB-T: conducted the experiments and helped write the manuscript. ZD: critical reading of the manuscript. EB: conducted experiments and analyzed data. OE: designed and performed experiments, analyzed data, and critical reading of the manuscript. NZ and OM: analyzed data and critical reading of the manuscript. EM: project supervision and critical reading. ER: project supervision, conceived, designed the experiments, and critical reading of the manuscript. All authors read and approved the final manuscript.

## Funding

This study was supported by the Israeli Ministry of Health.

## Conflict of Interest

The authors declare that the research was conducted in the absence of any commercial or financial relationships that could be construed as a potential conflict of interest.

## Publisher's Note

All claims expressed in this article are solely those of the authors and do not necessarily represent those of their affiliated organizations, or those of the publisher, the editors and the reviewers. Any product that may be evaluated in this article, or claim that may be made by its manufacturer, is not guaranteed or endorsed by the publisher.

## References

[1] Chan JF Kok KH Zhu Z Chu H To KK Yuan S . Genomic characterization of the 2019 novel human-pathogenic coronavirus isolated from a patient with atypical pneumonia after visiting Wuhan. Emerg Microbes Infect. (2020) 9:221–36. 10.1080/22221751.2020.1719902 31987001 PMC7067204

[2] Wu F Zhao S Yu B Chen YM Wang W Song ZG . A new coronavirus associated with human respiratory disease in China. Nature. (2020) 579:265–9. 10.1038/s41586-020-2008-3 32015508 PMC7094943

[3] Zhou P Yang XL Wang XG Hu B Zhang L Zhang W . A pneumonia outbreak associated with a new coronavirus of probable bat origin. Nature. (2020) 579:270–3. 10.1038/s41586-020-2012-7 32015507 PMC7095418

[4] Zhu N Zhang D Wang W Li X Yang B Song J . A Novel Coronavirus from patients with pneumonia in China, 2019. N Engl J Med. (2020) 382:727–33. 10.1056/NEJMoa2001017 31978945 PMC7092803

[5] Alm E Broberg EK Connor T Hodcroft EB Komissarov AB Maurer-Stroh S . Geographical and temporal distribution of SARS-CoV-2 clades in the WHO European Region, January to June 2020. Euro Surveill. (2020) 25:2001410. 10.2807/1560-7917.ES.2020.25.32.2001410 32794443 PMC7427299

[6] Korber B Fischer WM Gnanakaran S Yoon H Theiler J Abfalterer W . Tracking changes in SARS-CoV-2 spike: evidence that D614G increases infectivity of the COVID-19 virus. Cell. (2020) 182:812–27. 10.1016/j.cell.2020.06.043 32697968 PMC7332439

[7] Mercatelli D Giorgi FM. Geographic and genomic distribution of SARS-CoV-2 mutations. Front Microbiol. (2020) 11:1800. 10.3389/fmicb.2020.01800 32793182 PMC7387429

[8] Furuse Y. Genomic sequencing effort for SARS-CoV-2 by country during the pandemic. Int J Infect Dis. (2020) 103:305–7. 10.1016/j.ijid.2020.12.034 33333251 PMC7832795

[9] Donoghue M Hsieh F Baronas E Godbout K Gosselin M Stagliano N . A novel angiotensin-converting enzyme-related carboxypeptidase (ACE2) converts angiotensin I to angiotensin 1-9. Circ Res. (2000) 87:E1–9. 10.1161/01.RES.87.5.e1 10969042

[10] Huang Y Yang C Xu XF Xu W Liu SW. Structural and functional properties of SARS-CoV-2 spike protein: potential antivirus drug development for COVID-19. Acta Pharmacol Sin. (2020) 41:1141–9. 10.1038/s41401-020-0485-4 32747721 PMC7396720

[11] Letko M Marzi A Munster V. Functional assessment of cell entry and receptor usage for SARS-CoV-2 and other lineage B betacoronaviruses. Nat Microbiol. (2020) 5:562–9. 10.1038/s41564-020-0688-y 32094589 PMC7095430

[12] Li MY Li L Zhang Y Wang XS. Expression of the SARS-CoV-2 cell receptor gene ACE2 in a wide variety of human tissues. Infect Dis Poverty. (2020) 9:45. 10.1186/s40249-020-00662-x 32345362 PMC7186534

[13] Walls AC Park YJ Tortorici MA Wall A McGuire AT Veesler D. Structure, function, and antigenicity of the SARS-CoV-2 spike glycoprotein. Cell. (2020) 183:1735. 10.1016/j.cell.2020.11.032 33306958 PMC7833104

[14] Yan R Zhang Y Li Y Xia L Guo Y Zhou Q. Structural basis for the recognition of SARS-CoV-2 by full-length human ACE2. Science. (2020) 367:1444–8. 10.1126/science.abb2762 32132184 PMC7164635

[15] Liu S Xiao G Chen Y He Y Niu J Escalante CR . Interaction between heptad repeat 1 and 2 regions in spike protein of SARS-associated coronavirus: implications for virus fusogenic mechanism and identification of fusion inhibitors. Lancet. (2004) 363:938–47. 10.1016/S0140-6736(04)15788-7 15043961 PMC7140173

[16] Podder S Ghosh A Ghosh T. Mutations in membrane-fusion subunit of spike glycoprotein play crucial role in the recent outbreak of COVID-19. J Med Virol. (2020). 10.1002/jmv.26598 33090493 PMC7675664

[17] van Dorp L Acman M Richard D Shaw LP Ford CE Ormond L . Emergence of genomic diversity and recurrent mutations in SARS-CoV-2. Infect Genet Evol. (2020) 83:104351. 10.1016/j.meegid.2020.104351 32387564 PMC7199730

[18] Wrobel AG Benton DJ Xu P Calder LJ Borg A Roustan C . Structure and binding properties of pangolin-CoV spike glycoprotein inform the evolution of SARS-CoV-2. Nat Commun. (2021) 12:837. 10.1038/s41467-021-21006-9 33547281 PMC7864994

[19] Wrobel AG Benton DJ Xu P Roustan C Martin SR Rosenthal PB . SARS-CoV-2 and bat RaTG13 spike glycoprotein structures inform on virus evolution and furin-cleavage effects. Nat Struct Mol Biol. (2020) 27:763–7. 10.1038/s41594-020-0468-7 32647346 PMC7610980

[20] Di Giorgio S Martignano F Torcia MG Mattiuz G Conticello SG. Evidence for host-dependent RNA editing in the transcriptome of SARS-CoV-2. Sci Adv. (2020) 6:eabb5813. 10.1126/sciadv.abb5813 32596474 PMC7299625

[21] Robson F Khan KS Le TK Paris C Demirbag S Barfuss P . Coronavirus RNA proofreading: molecular basis and therapeutic targeting. Mol Cell. (2020) 80:1136–8. 10.1016/j.molcel.2020.11.048 33338403 PMC7833706

[22] Yao H Lu X Chen Q Xu K Chen Y Cheng M . Patient-derived SARS-CoV-2 mutations impact viral replication dynamics and infectivity in vitro and with clinical implications in vivo. Cell Discov. (2020) 6:76. 10.1038/s41421-020-00226-1 33298872 PMC7595057

[23] Lorenzo-Redondo R Nam HH Roberts SC Simons LM Jennings LJ Qi C . A clade of SARS-CoV-2 viruses associated with lower viral loads in patient upper airways. EBioMedicine. (2020) 62:103112. 10.1016/j.ebiom.2020.103112 33186810 PMC7655495

[24] Muller NF Wagner C Frazar CD Roychoudhury P Lee J Moncla LH . Viral genomes reveal patterns of the SARS-CoV-2 outbreak in Washington State. Sci Transl Med. (2021) 13:eabf0202. 10.1126/scitranslmed.abf0202 33941621 PMC8158963

[25] Volz E Hill V McCrone JT Price A Jorgensen D O'Toole A . Evaluating the Effects of SARS-CoV-2 Spike Mutation D614G on Transmissibility and Pathogenicity. Cell (2021) 184:64–75. 10.1016/j.cell.2020.11.020 33275900 PMC7674007

[26] Sette A Crotty S. Adaptive immunity to SARS-CoV-2 and COVID-19. Cell. (2021) 18:861–80. 10.1016/j.cell.2021.01.007 33497610 PMC7803150

[27] Krammer F. SARS-CoV-2 vaccines in development. Nature. (2020) 586:516–27. 10.1038/s41586-020-2798-3 32967006

[28] Tegally H Wilkinson E Giovanetti M Iranzadeh A Fonseca V Giandhari J . Detection of a SARS-CoV-2 variant of concern in South Africa. Nature (2021) 592:438–43. 10.1038/s41586-021-03402-9 33690265

[29] Tang JW Tambyah PA Hui DS. Emergence of a new SARS-CoV-2 variant in the UK. J Infect. (2020) 82:e27–8. 10.1016/j.jinf.2020.12.024 33383088 PMC7834693

[30] Kirby T. New variant of SARS-CoV-2 in UK causes surge of COVID-19. Lancet Respir Med. (2021) 9:e20–1. 10.1016/S2213-2600(21)00005-9 33417829 PMC7784534

[31] Starr TN Greaney AJ Hilton SK Ellis D Crawford KHD Dingens AS . Deep mutational scanning of SARS-CoV-2 receptor binding domain reveals constraints on folding and ACE2 binding. Cell. (2020) 182:1295–310. 10.1016/j.cell.2020.08.012 32841599 PMC7418704

[32] Wise J. Covid-19: the E484K mutation and the risks it poses. BMJ. (2021) 372:n359. 10.1136/bmj.n359 33547053

[33] Duarte CM Jamil T Gojobori T Alam I. Detection of SARS-CoV-2 variants requires urgent global coordination. Int J Infect Dis. (2021) 109:50–3. 10.1016/j.ijid.2021.06.027 34147667 PMC8240518

[34] Gohl DM Garbe J Grady P Daniel J Watson RHB Auch B . A rapid, cost-effective tailed amplicon method for sequencing SARS-CoV-2. BMC Genomics. (2020) 21:863. 10.1186/s12864-020-07283-6 33276717 PMC7716288

[35] Charre C Ginevra C Sabatier M Regue H Destras G Brun S . Evaluation of NGS-based approaches for SARS-CoV-2 whole genome characterisation. Virus Evol. (2020) 6:veaa075. 10.1093/ve/veaa075 33318859 PMC7665770

[36] Zuckerman NS Pando R Bucris E Drori Y Lustig Y Erster O . Comprehensive analyses of SARS-CoV-2 transmission in a public health virology laboratory. Viruses. (2020) 12:854. 10.3390/v12080854 32764372 PMC7472171

[37] Nissan I Freidlin PJ Goldblat D Kaidar-Shwartz H Dveyrin Z Grossman R et al. Molecular Typing of Microbes. (2018). USA patent: 20210355526.

[38] Baker DJ Aydin A Le-Viet T Kay GL Rudder S de Oliveira Martins L . CoronaHiT: high-throughput sequencing of SARS-CoV-2 genomes. Genome Med. (2021) 13:21. 10.1186/s13073-021-00839-5 33563320 PMC7871948

[39] Huang J Zhao L. A high-throughput strategy for COVID-19 testing based on next-generation sequencing. medRxiv [Preprint]. (2020). 10.1101/2020.06.12.20129718 32588005 PMC7310665

[40] Itokawa K Sekizuka T Hashino M Tanaka R Kuroda M. Disentangling primer interactions improves SARS-CoV-2 genome sequencing by multiplex tiling PCR. PLoS ONE. (2020) 15:e0239403. 10.1371/journal.pone.0239403 32946527 PMC7500614

