# Supplementary material for: HiSpike Method for High-Throughput Cost Effective Sequencing of the SARS-CoV-2 Spike Gene
Source: Front Med (Lausanne). 2022 Jan 11;8:798130. doi: 10.3389/fmed.2021.798130 (PMC8787038; doi:10.3389/fmed.2021.798130)

HiSpike method for high-throughput cost effective sequencing of the SARS-CoV-2 spike gene

Fass Ephraim[1], Zizelski Valenci Gal[1], Rubinstein Mor[1], Freidlin Paul J[1], Rosencwaig Shira[1], Kutikov Inna[1], Werner Robert[1], Ben-Tovim Nofar[1], Bucris Efrat[2], Erster Oran[2], Zuckerman Neta S[2], Mor Orna[2,3], Mendelson Ella[2,3], Dveyrin Zeev[1], Rorman Efrat[1], and Nissan Israel[1,*]

[1]National Public Health Laboratory, Public Health Services, Ministry of Health, Tel Aviv, Israel

[2]Central Virology Laboratory, Public Health Services, Ministry of Health, Chaim Sheba Medical Center, Tel-Hashomer, Ramat Gan, Israel

[3]School of Public Health, Sackler faculty of Medicine, Tel Aviv University, Tel Aviv, Israel

[*]Corresponding author: israel.nissan@moh.gov.il

Supplementary file S6. Comparison between spike sequence breadth obtained by HiSpike and ARTIC methods. A. Spike gene sequences of 90 samples were divided to 4 groups based on their Ct values (15-20, 20-25, 25-30, and 30-35). ARTIC results are presented in black and HiSpike in gray. The percentage of spike gene coverage, with at least 5 reads, at different Ct levels is shown by Boxplots. The Boxplots represent the coverage breadth value distributions among samples from the lower to the upper quartiles. The inner horizontal line indicates the median. The vertical whiskers extend to the most extreme data point which is no more than 1.5 times the interquartile range. More extreme data points are drawn as circles. B. plot of the number of samples (Y axis) with a 5 read cover or more at each position along the spike gene (X axis). ARTIC method (solid line) and HiSpike method (dotted line). The region encoding the RBD is marked (red bar) for reference.

Supplementary file S6

A

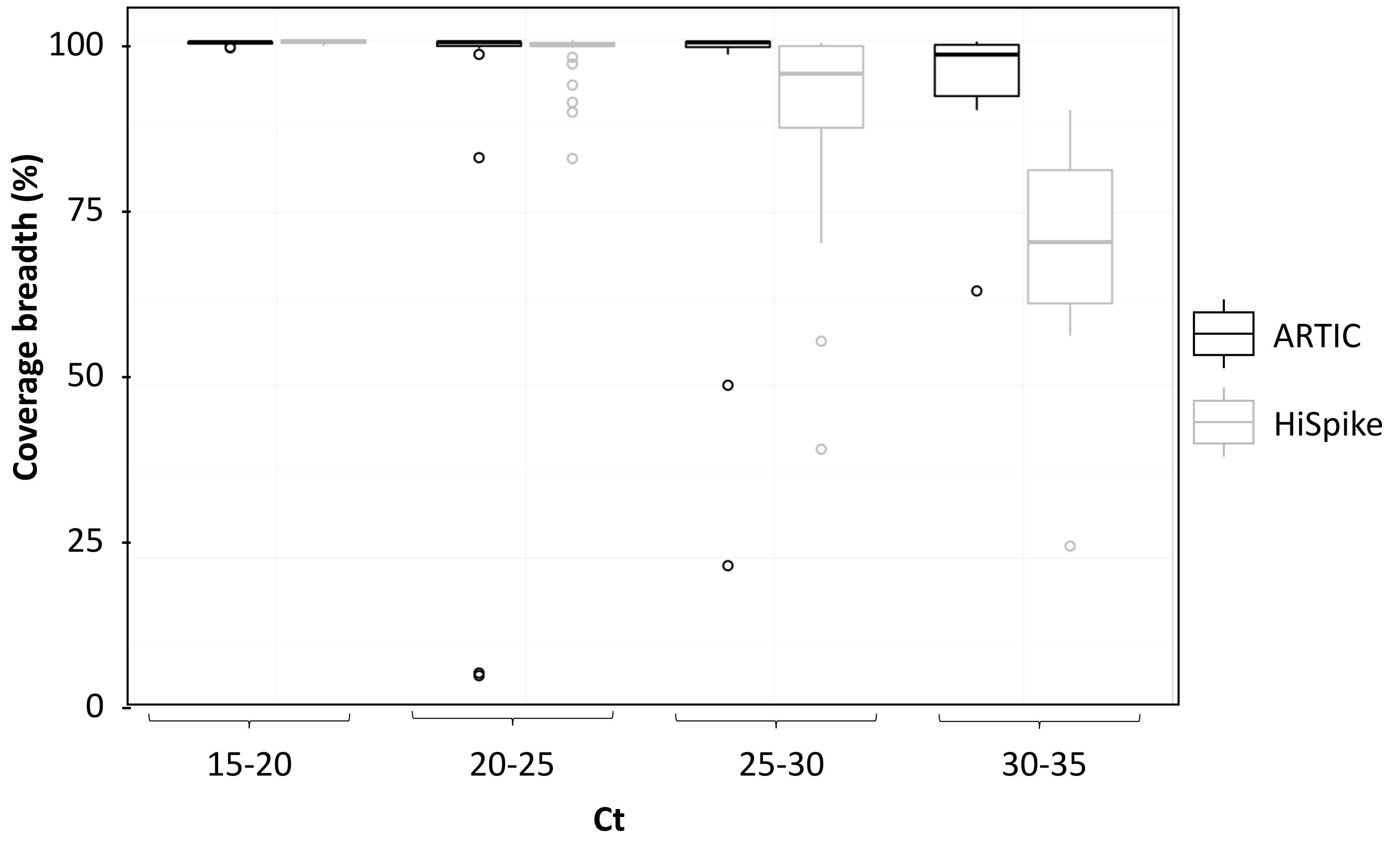

B

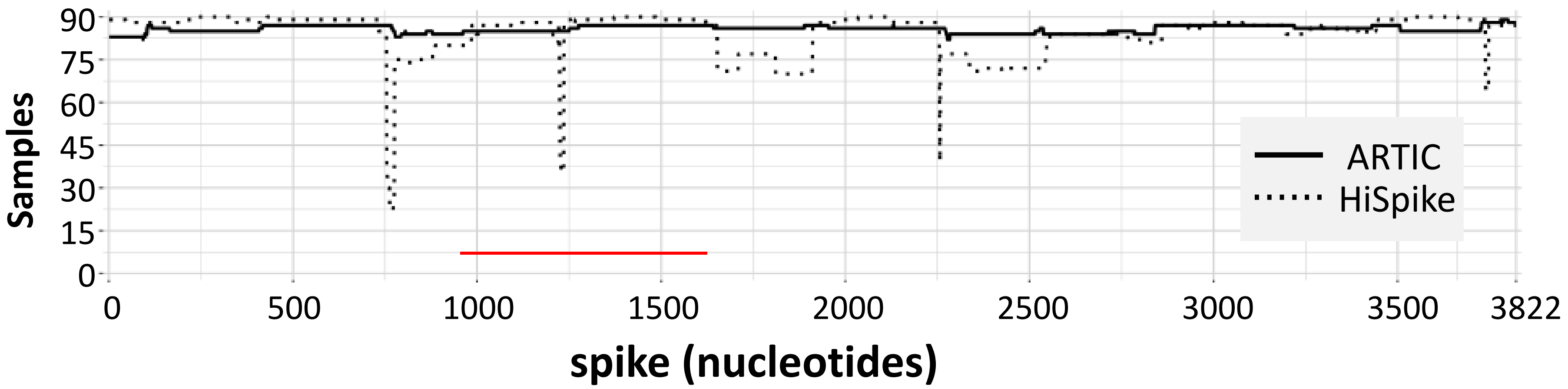

Supplement: Supplementary file 6 [file Data_Sheet_6.pdf]
